# Mesoporous Ag@WO_3_ core–shell, an investigation at different concentrated environment employing laser ablation in liquid

**DOI:** 10.1038/s41598-024-55146-x

**Published:** 2024-03-05

**Authors:** Evan T. Salim, Jehan A. Saimon, Maryam S. Muhsin, Makram A. Fakhri, Mustafa H. Amin, Ahmad S. Azzahrani, Raed Khalid Ibrahim

**Affiliations:** 1 Applied Science Department, University of Technology-Iraq, Baghdad, Iraq; 2 Laser and Optoelectronic Department, University of Technology-Iraq, Baghdad, Iraq; 3 Institute of Laser for Postgraduate Studies, University of Baghdad, Baghdad, Iraq; 4 Electrical Engineering Department, Northern Border University, Arar, Saudi Arabia; 5 Al-Farahidi University, Baghdad, Iraq

**Keywords:** Ag NPs, WO_3_ NPs, Core–shell, Nanoparticles, Laser ablation, PLAL, Materials science, Nanoscience and technology

## Abstract

In this study, silver-tungsten oxide core–shell nanoparticles (Ag–WO_3_ NPs) were synthesized by pulsed laser ablation in liquid employing a (1.06 µm) Q-switched Nd:YAG laser, at different Ag colloidal concentration environment (different core concentration). The produced Ag–WO_3_ core–shell NPs were subjected to characterization using UV–visible spectrophotometry, X-ray diffraction (XRD), transmission electron microscopy (TEM), energy-dispersive spectroscopy, electrical analysis, and photoluminescence PL. The UV–visible spectra exhibited distinct absorption peaks at around 200 and 405 nm, which attributed to the occurrence of surface Plasmon resonance of Ag NPs and WO_3_ NPs, respectively. The absorbance values of the Ag–WO_3_ core–shell NPs increased as the core concentrations rose, while the band gap decreased by 2.73–2.5 eV, The (PL) results exhibited prominent peaks with a central wavelength of 456, 458, 458, 464, and 466 nm. Additionally, the PL intensity of the Ag–WO_3_-NP samples increased proportionally with the concentration of the core. Furthermore, the redshift seen at the peak of the PL emission band may be attributed to the quantum confinement effect. EDX analysis can verify the creation process of the Ag–WO_3_ core–shell nanostructure. XRD analysis confirms the presence of Ag and WO_3_ (NPs). The TEM images provided a good visualization of the core-spherical shell structure of the Ag–WO_3_ core–shell NPs. The average size of the particles ranged from 30.5 to 89 (nm). The electrical characteristics showed an increase in electrical conductivity from (5.89 × 10^−4^) (Ω cm)^−1^ to (9.91 × 10^−4^) (Ω cm)^−1^, with a drop in average activation energy values of (0.155 eV) and (0.084 eV) at a concentration of 1.6 μg/mL of silver.

## Introduction

Core–shell nanocomposites (NCs) have garnered significant interest in recent years due to their exceptional structural, chemical, photocatalytic, physical, and optical characteristics^1–7^. Core–shell NCs have a significant coupling exciton effect that exists between the core (noble metal) with surface plasmon resonance (SPR) characteristics and the excitons of the semiconductors (shell)^8–10^. A variety of methods have been documented for producing core–shell NPs. The wet chemistry approach involves the interaction between the core or shell material and the surrounding liquid, resulting in the formation of core–shell structures. Although this process does produce a substantial outcome, it is occasionally deemed unsustainable due to the use of hazardous substances. In contrast, physical approaches provide several benefits in comparison to wet chemical procedures. Additionally, physical approaches need little sample preparation and do not necessitate the use of environmentally toxic chemicals. The physical techniques include several approaches, such as heat flow tubes, flame assembly, spray pyrolysis techniques, and plasma procedures^11^. The aforementioned techniques are implemented to generate core–shell nanoparticles in a relatively brief amount of time. Laser ablation is regarded as one of the physical methods within this context. Laser ablation has been used in many methodologies for the synthesis of core–shell nanoparticles, such as laser ablation in the presence of a reactive gas and laser ablation in a liquid medium^12^. Yellow powdered tungsten oxide (WO3) has a density of 7.16 g/cm^3^, a melting point of 1473 °C, and a boiling temperature of 1700 °C. While it is insoluble in water and most acids (apart from hydrofluoric acid), it is soluble in ammonium hydroxide, where it forms tungstate^13–17^. Tungsten oxide (WO_3_) has a narrow bandgap ranging from 2.7 to 3.1 (eV), making it a material with notable reactivity within the visible light range. Tungsten trioxide (WO_3_) exhibits stable semiconductor properties^18,19^. Due to its unusual physicochemical characteristics, it has the potential to be used in a variety of technical domains, including lithium-ion batteries^20,21^, solar energy devices, photocatalysts^16,22^, smart windows, electronic information displays and electrochromic devices^23–29^. While WO3, ZnO, and SnO are commonly employed as gas sensor applications, Tungsten, specifically, has the capability to detect gases that are toxic and destructive, such as NO2, Acetone, and NH3^16,30^, H2S, and hydrocarbons such as carbon monoxide, such as benzene and methane^31–33^. Thus, the production of WO3 is important. However, WO3 is not widely available due to price rises and export limitations imposed by manufacturing countries^34^. The extremely common semiconductor metal oxides used in optoelectronic devices include WO3, MoO3, SnO2, TiO2, and ZnO. Surprisingly, among all these, WO3 is used in many electronic devices due to its tunable properties of high thermal stability, visible-range optical absorption, surface morphology, and chemical composition^35,36^. Extensive research has been conducted on the plasmonic properties shown by silver (Ag) NPs, primarily owing to their exceptional performance relative to other surface plasmon resonance (SPR) metals throughout the visible region of the electromagnetic spectrum. Silver (Ag) nanostructures exhibit distinct, strong, and precise plasmonic phenomena within the visible region of the electromagnetic spectrum. Moreover, localized surface plasmon resonance (LSPR) may manifest in silver (Ag) nanostructures measuring less than 5 nm in size^37–40^. The core–shell nanostructures provide many benefits. By altering the material composition, core/shell size ratio, and the surrounding medium, it is possible to adjust and combine the plasmonic response of the nanoparticles. The control of nanostructured materials has garnered significant attention in recent years due to the possible properties that may be achieved and manufactured. These materials possess versatile properties that make them suitable for several application domains, including but not limited to biological, optical, magnetic electronic fields and electrical^10,41–49^. Numerous fields of study have developed an interest in nanoscience. Consequently, it is essential to produce nanoparticles with the ideal size distribution, morphology, and crystallinity^50^. Many controlled synthesis techniques for Ag-NPs, including biosynthesis, evaporation condensation, sol–gel, laser ablation, microwave processing, electron irradiation, electrochemical and photochemical have been documented^51,52^. Every approach has both benefits and drawbacks^53–57^. Nevertheless, every technique can be categorized into one of two methodologies: bottom-up or top-down. Recent studies have shown that the pulsed laser ablation in liquid medium technique (PLAL) is an effective and promising method for regulating the particle size of nanostructured materials that are manufactured^58^. The underlying principle of this technique is the use of high-intensity pulsed laser ablation to initiate a laser-matter interaction on the surface of the object under examination. This interaction results in the generation of a plasma plume that is fully submerged in a liquid medium^59–65^. This phenomenon results in the establishment of a unique thermodynamic condition characterized by elevated pressure levels. The generation of nanoscale particles is facilitated by the creation of distinctive thermodynamic conditions characterized by elevated levels of pressure and temperature^64,66^. The PLAL processes have the potential to generate nanostructured materials in an extensive range of shapes and dimensions. PLAL, which stands for metal or metal oxide-NCs, has recently been acknowledged as the most adaptable, promising, and straightforward technique for producing such materials. An instance of the effective use of the Nd:YAG laser is the creation of Ag nanoparticle-decorated carbon nanotubes (CNTs) to enhance the photocatalytic efficacy in the removal of naphthalene from^67–79^ water^80^ and MWCNTs/Ag NCs for catalytic degradable material for nitro compounds and dyes^81^. The PLAL process has many benefits, including simplicity, low cost, a decrease in by-product generation, the absence of catalyst and vacuum requirements, high-purity nanomaterial manufacturing, and precise control over particle shape and size by modifying laser settings. The metal-oxide core–shell architecture offers several benefits in terms of enhanced stability against sintering^82^ and leaching^83^, the potential to impart magnetic properties to the NCs^84,85^, and, notably, the ability to modify the material's electronic structure through metal-oxide interactions^86^. Charge transfer is a critical factor in determining the physicochemical, spectroscopic, and catalytic characteristics of the NCs materials^87–89^. By modifying their structure (e.g., shell thickness and core size) and composition (e.g., alloying the core or doping the shell), metal-oxide Nc can enable one to adjust their properties due to the multitude of interactions observed within them^90–92^. Consequently, the characteristics of metal-oxide core–shell NCs are unlike those of any other material^93,94^. The Ag–WO_3_ NPs are of particular interest for several reasons. Firstly, the Ag (NP) core can effectively generate strong localized surface plasmons (LSP) across the visible spectrum. Secondly, the LSP can be optimized by adjusting the core (Ag-NPs) diameter. Thirdly, WO_3_ itself is an excellent gas sensor material. Fourthly, the Ag–WO_3_ core–shell NPs are expected to function as a good Schottky junction sensor. Fifthly, the well-known layered structure of WO_3_ at nanometer thickness may enhance the sensor performance through quantum effects. Lastly, the combination of the core (Ag-NPs) and the layered WO_3_ shell could lead to the development of a new generation of gas sensors with enhanced quantum effects. This work aimed to produce Ag–WO_3_ core–shell nanoparticles utilizing a laser ablation approach in deionized water at normal room temperature. The XRD, UV–Vis spectrum, PL, EDX, FE-SEM, and TEM techniques were used to analyze the structure, morphology, optical, and electrical features of Ag–WO_3_ core–shell NPs generated at different core concentrations. Additionally, the room-temperature electrical properties of these NPs were also examined.

As a novel contribution, this work presents a manipulation and fine-tuning of the optical and electrical properties of Ag–WO_3_ core–shell nanoparticles by varying the Ag core concentration employing laser ablation in liquid technique, the study showcased the impact of core concentration on the structural, morphological, optical, and electrical characteristics of Ag–WO_3_ core–shell nanoparticles.

## Experimental work

**Figure 1:**
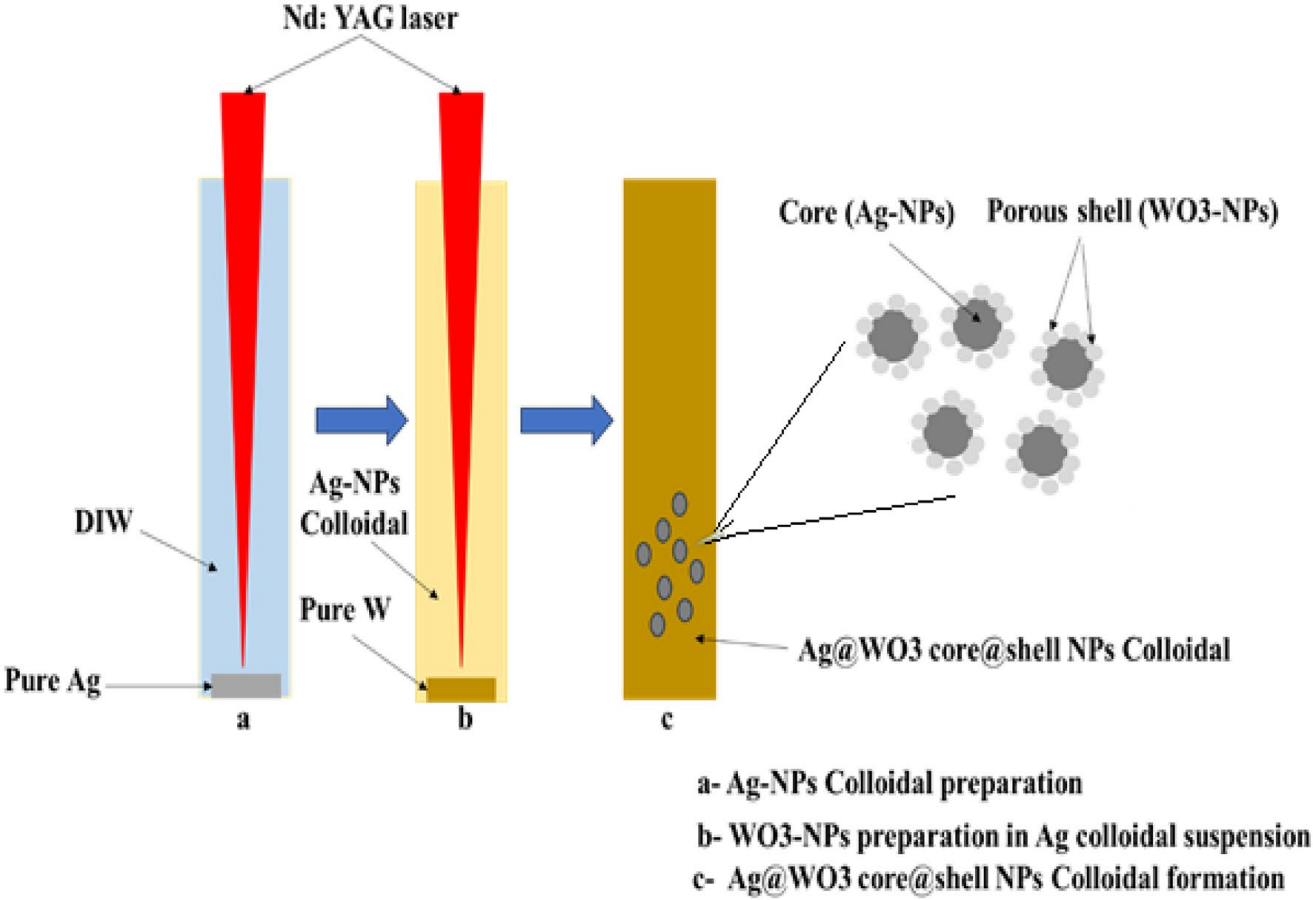
Schematic diagram illustrating the process of forming Ag–WO_3_ core-Porous shell NPs using pulsed laser ablation in water.

**Figure 2:**
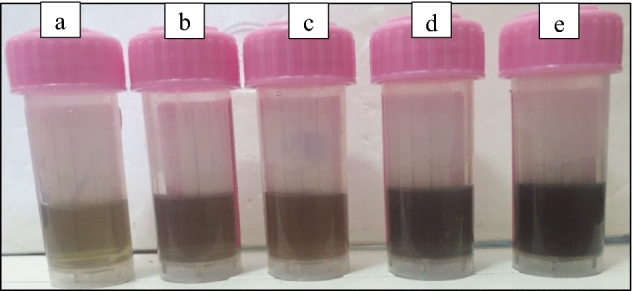
Photograph of freshly prepared colloidal Ag–WO_3_ core–shell NPs at different concentrations of Ag (a—0.36, b—0.76, c—1.2, d—1.6, e—1.96) μg/mL.

We have synthesized Ag–WO_3_ core–shell NPs by a two-step process of laser ablation in water. Initially, a 1064 nm Nd:YAG pulsed laser beam was used to ablate a square silver target plate with dimensions of (0.8*0.8 mm) and high purity of (99.9%) that was submerged in 3 ml of distilled water inside a glass container without the use of any chemical additives. A repetition rate of 1 Hz, 10 focal length of the lens and a pulse width of 15 ns were used. The silver target was cleaned before immersion and irradiation by dipping it in acetone and washing it in pure water. The laser ablation procedure was conducted on the Ag target using a laser fluence of 6.12 J/cm^2^ and number of laser pulses (200, 250, 300, 350, and 400). The experiment was conducted at different concentrations of silver. Resulting in the production of nanoparticles in the form of a suspension (0.36, 0.76, 1.2, 1.6, and 1.96 μg/mL). Step two, the silver target was subsequently substituted for a tungsten target in the solution, to produce the Ag–WO_3_ colloidal core–shell. Using 1064 nm, 1 Hz, 10 focal length lens with a constant laser fluence of 76.34 J/cm^2^ laser pulses (300), respectively. Figure 1 illustrates the schematic diagram depicting the process of forming Ag–WO_3_ core-Porous shell NPs by laser ablation in water (Table 1). Figure 2 depicts an image of newly formed colloidal Ag–WO_3_ core–shell NPs. It is evident that increasing the number of laser pulses caused an increase in core concentrations and change in the color of the solution, transitioning from light yellow to deep yellow. This change in color indicates a variation in particle size according to the number of laser pulses. The concentrations of the Ag colloidal nanoparticles were determined by estimating the weight of the Ag target before and after ablation by laser. The concentration was estimated as a function of the laser pulses number using a five digit (digital scale) precision weighing instrument that can measure weight to an accuracy of 0.00001 g. The following formulae were used. Five distinct concentrations of (Ag) nanoparticles where used^95–97^.

1 $$\Delta {\text{M}} = \left( {{\text{m}}_{1} - {\text{m}}_{2} } \right)\,\upmu {\text{g}}$$

where: m_1_ and m_2_ denote the target's mass prior to and subsequent to ablation, respectively. The formula for calculating the concentration is as follows^98–100^:

2 $${\text{C}} = \Delta {\text{M}}\left( {\upmu {\text{g}}} \right)/{\text{V}}\left( {{\text{m}}_{{\text{l}}} } \right)$$

where: V stands for the liquid’s amount.

**Table 1:** Explained different concentrations of colloidal silver versus number of laser pulses.

Sample	Laser pulses	Concentrations (μg/mL)
1	200	0.36
2	250	0.76
3	300	1.2
4	350	1.6
5	400	1.96

In order to study the structural characteristics of Ag–WO_3_ core–shell NPs deposited on silicon substrate, XRD measurement was used (XRD-6000, Shimadzu, X-ray diffractometer). In this work, all samples were processed using the FE-SEM (ARYA Electron Optic) equipment, which featured an energy dispersive X-ray (EDX), to accomplish the advantages indicated before. The TEM (type CM10 pw6020, Philips-Germany) was used to analyze the size and form of Ag–WO_3_ core–shell NPs. Using a UV–Vis double beam spectrophotometer (Shimadzu UV-1800), the optical absorbance of the colloidal nanoparticles solution was documented. We measured the resistance (R) values of the Ag–WO_3_ core–shell samples using a Kiethly electrometer and an excitation wavelength of 325 nm, as part of the Pl analysis.

## Results and discussion

**Figure 3:**
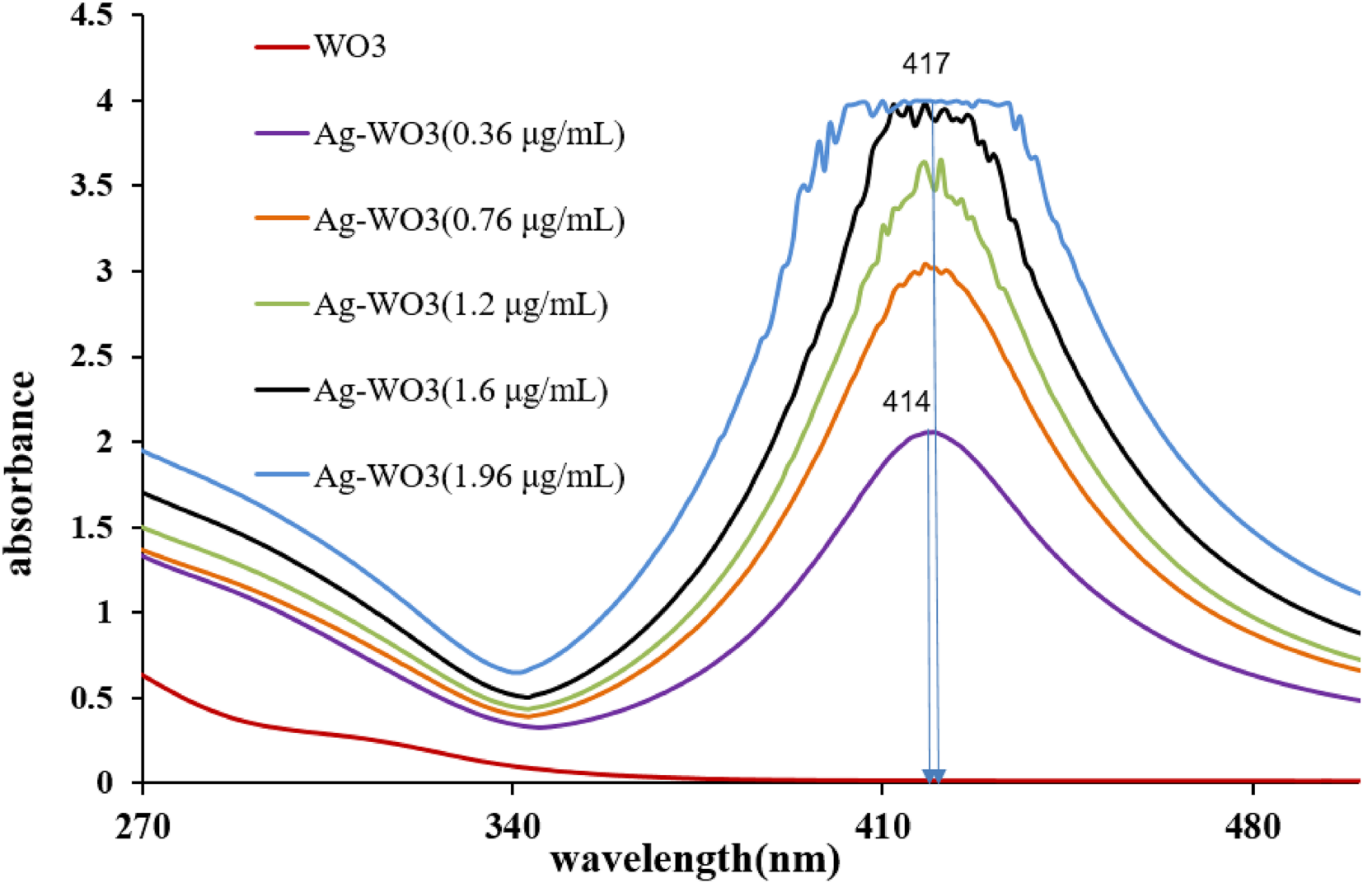
Absorption spectrum of Ag–WO_3_ nanoparticles with various Ag nanoparticle concentrations.

**Figure 4:**
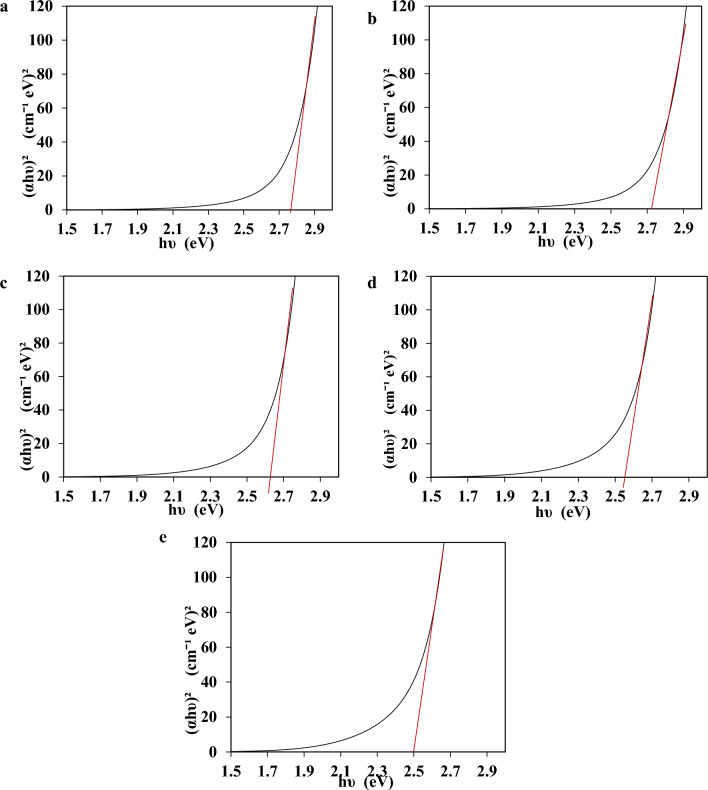
The absorption and energy gap spectra of Ag–WO_3_-NPs at different concentrations of Ag (**a**—0.36, **b**—0.76, **c**—1.2, **d**—1.6, **e**—1.96) μg/mL.

The optical properties and the energy gap of the Ag–WO_3_ core–shell NPs were determined by means of ultraviolet spectroscopy. The absorption spectrum of Ag–WO_3_ core–shell NPs samples was obtained in Fig. 3 using a fluence of 76.43 J/cm^2^, 400 laser pulses, and a wavelength of 1064 nm. The samples were prepared with varying concentrations of silver (0.36, 0.76, 1.2, 1.6, and 1.96 μg/mL) and a constant fluence of 6.12 J/cm^2^. Figure 3 shows the optical absorption spectra of the Ag–WO_3_ core–shell nanoparticles that were produced by employing pulse laser ablation in water. The number of laser pulses used in the synthesis process varied between 200, 250, 300, 350, and 400. A strong plasmonic absorption peak at 410 nm is easily discernible in the absorption spectra of Ag–WO_3_ core–shell nanoparticles. The spectrum exhibited distinct and broad absorption peaks in the visible region, located at approximately 414, 416, 415, 417, and 417 nm for the samples with concentrations of 0.36, 0.76, 1.2, 1.6, and 1.96 μg/mL, respectively. In agreement with one of the previous studies^101,102^. The intensities of all distinctive peaks, which were seen in the Ag–WO_3_ NPs samples, exhibit an increase when the amounts of silver particles are increased. The observed peaks ascribed to the presence of Ag-NPs. Furthermore, it was observed that there were minor absorption peaks at around 311, 312, 313, 314, and 315 nm, indicating the presence of WO_3_ nanoparticles for the samples with concentrations of 0.36, 0.76, 1.2, 1.6, and 1.96 μg/mL, respectively. After the incorporation of Ag NPs into WO_3_ NPs, the absorption band edge of bare WO_3_ is redshifted (toward a longer wavelength). The presence of both peaks provided evidence for the creation of the Ag–WO_3_ core shell NPs^103–105^. This result gives a clear indication of the effect of Ag nanoparticles due to the effect of SPR coming from Ag (core) nanoparticles. Consequently, the bandgap energy of the material decreased as a result of the redshifted of absorption band edges produced by the incorporations of Ag NPs. The band gaps of the samples at different concentrations of silver (0.36, 0.76, 1.2, 1.6, and 1.96 μg/mL) are presented in Fig. 4. The optical absorption band gap follows a power law when incident photon energy is greater than the band gap and above the exponential^106–109^:

3 $$\left( {\alpha {\text{h}}\upnu } \right) = \beta \left( {{\text{h}}\upnu {-}E_{g} } \right)^{n}$$

where *E*_*g*_ is the optical bandgap, *α* is the absorption coefficient, *n* is an exponent, *β* is the edge with parameter and hʋ is the incident photon energy.

It can be observed from Fig. 4 that the optical band gap energy decreases to 2.75, 2.73, 2.62, 2.55, and 2.5 eV as the concentration of Ag increases. These values are very close to those stated in a previous study^110^, the band gap tailoring in core–shell NPs was attributed to its shape and the quantum confinement effect^111,112^. The interfaces of Ag–WO_3_ NPs have a significant impact on the processes of charge transfer and separation^113,114^. This is attributed to the presence of Ag NPs, which function as localized surface plasmons (LSP). This process credits the combination of electromagnetic waves to the oscillations of electrons^115^. This procedure enables the reduction of the band gap in the core cell structure. As a result, the adjusted band gap allows for increased interaction between visible light and the core–shell compared to the regulated WO_3_. The core–shell has promising optical and electrical properties because to its ability to fine-tune the band gap features^116,117^. Consequently, the bandgap energy of the material decreased as a result of the redshifted of absorption band edges produced by the incorporations of Ag NPs.

The flat peak in surface plasmon resonance typically refers to the collective oscillation of electrons at the interface between a metal and a dielectric material when excited by incident light. The resonance condition occurs when the momentum of incident photons matches the momentum of the surface plasmons.

The SPR response may not exhibit a sharp peak, making it challenging to identify the resonance position. This could be due to various factors, such as broadening of the SPR peak due to particle size distribution, polydispersity, or other experimental conditions.

**Figure 5:**
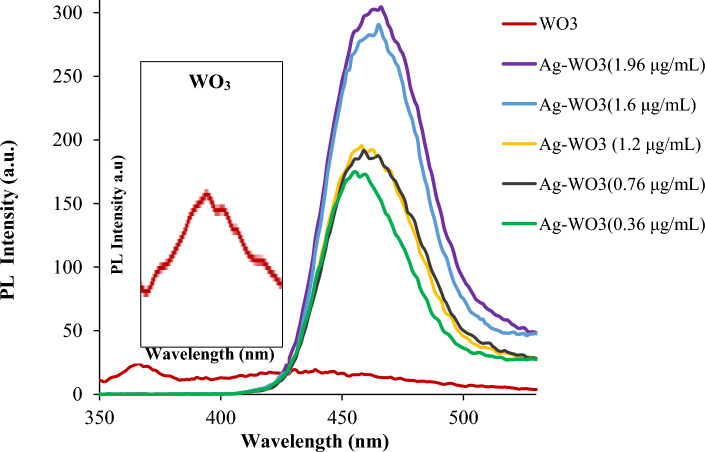
PL spectrum of Ag–WO_3_ nanoparticles with various Ag nanoparticle concentrations.

Figure 5 displays the photoluminescence (PL) spectrum of core–shell NPs consisting of silver (Ag) and tungsten oxide (WO_3_). The photoluminescence (PL) studies revealed increased intensities in all the Ag–WO_3_ samples created using varying amounts of silver (0.36, 0.76, 1.2, 1.6, and 1.96 μg/mL). The spectrum shows excitation bands located at 456, 458, 458, 464, and 466 nm, which correspond to energy gaps of about 2.71, 2.7, 2.7, 2.67, and 2.66 eV, respectively. The energy gap value approximated using PL data is marginally greater in magnitude compared to what was ascertained using UV–vis data^118^. Further, it differs from the energy gap of pure WO_3_. The PL intensities of the Ag–WO_3_ NPs peaks exhibited a significant increase compared to the PL peak of WO3. Additionally, these peaks show a minor shift, which may be ascribed to the enhanced photoluminescence seen in the NCs structure. This improvement is due to the incorporation of Ag-NPs. These results are consistent with UV–visible results. Ag–WO_3_ CS-NPs' emission energy and PL emission wavelength are displayed in Table 2 as a function of Ag concentrations.

**Table 2:** The Ag–WO_3_ core–shell NPs' emission energy and PL emission wavelength as a function of Ag concentrations.

Ag concentrations (μg/mL)	UV–vis energy gap (eV)	PL	
		Emission peak wavelength (nm)	Band gap energy (eV)
0.36	2.73	456	2.71
0.76	2.72	458	2.7
1.2	2.6	458	2.7
1.6	2.55	465	2.67
1.96	2.5	466	2.66

**Figure 6:**
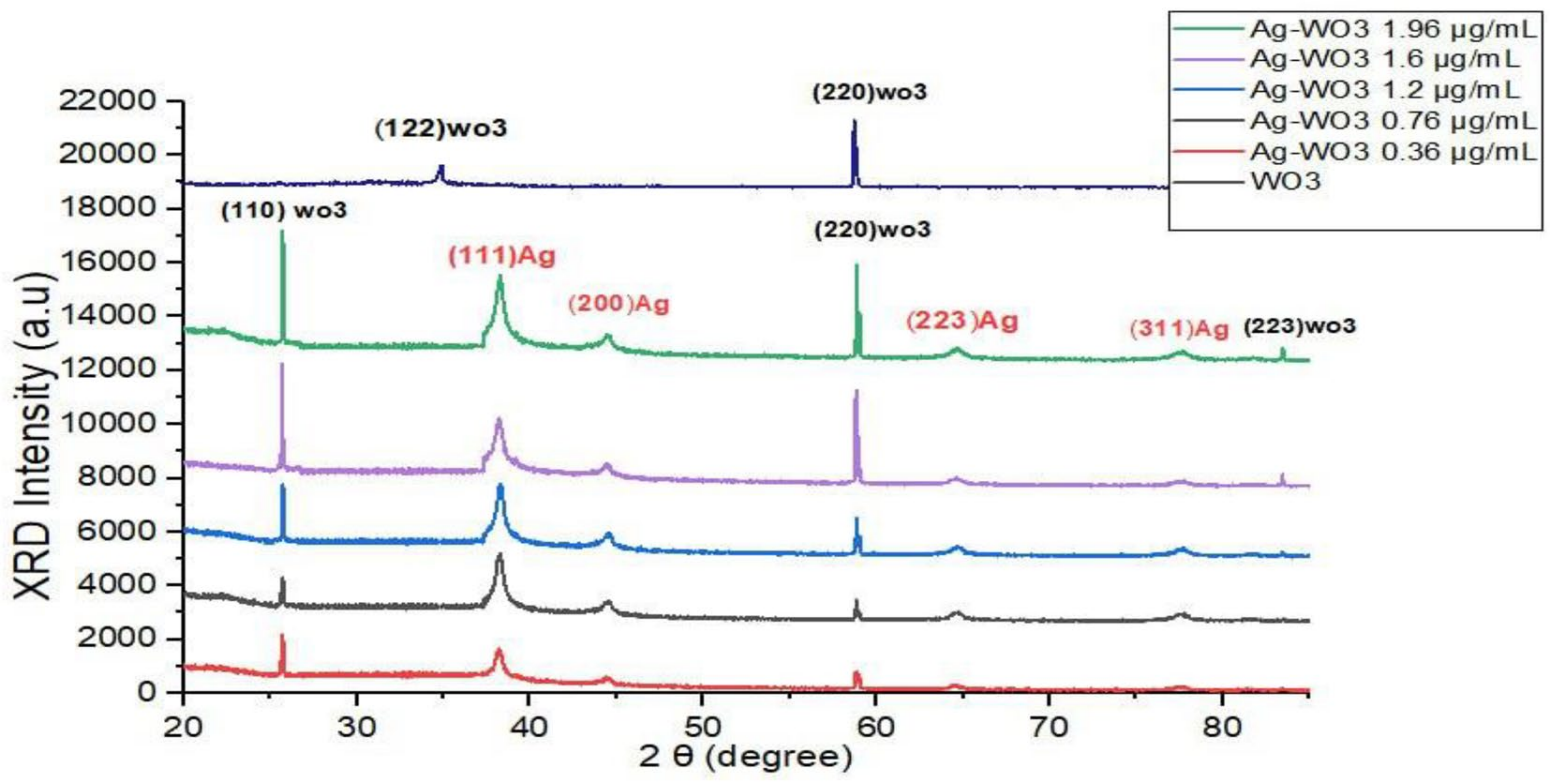
XRD patterns of Ag-–O_3_ nanoparticles using various Ag concentrations.

Figure 6 displays the diffraction peaks corresponding to the core–shell samples. These peaks were generated using a fluence of 76.34 J/cm^2^ and 400 laser pulses. The figure also displays a wavelength of 1064 nm with different concentrations of silver (0.36, 0.76, 1.2, 1.6, and 1.96 μg/mL). This figure further displays the prominent peaks that can be ascribed to hexagonal WO_3_, as indicated by the JCPDS card number 00-33-1387 and PDF number #75-2187. The peaks are located at 2θ values of 25.5° and 58.84°, corresponding to the orientations (110) and (220), respectively. On the other hand, the peak at 2θ = 83.4o with orientations (223), indexed to JCPDS card no. 89-1287, is related to the tetragonal WO_3_ phase. The presence of Ag-NPs was verified by the peaks at 2θ = 38.2o, 44.3o, 64.5o, and 77.86o with orientations (111), (200), (223), and (311). This can be attributed to cubic Ag nanoparticles, according to pdf number 870720. Due to the relatively high diameter of the core, significant diffraction peaks are generated. XRD identification of the core–shell configuration^119–121^ showed that these results are in line with the study^122^. When exposed to X-ray radiation, the greater concentrations of aggregated silver nanoparticles resulted in a greater degree of reflection^123,124^. For the WO_3_ sample, two peaks appeared at 2θ: 28.92° and 58.84°. While 28.92° corresponds to the (122) plane according to (JCPDS # 201323) triclinic WO_3_ phase structure, 58.84° corresponds to the (220) plane hexagonal WO3 phase structure. In addition, the XRD patterns of Ag–WO_3_ core/shell NPs exhibit a marginally greater intensity than those of shell NPs (Fig. 6). This may be the result of incorporation Ag core NPs causing an increase in particle size and crystallinity. Table 3 illustrates the grain size, dislocation densities, Miller indices, and micro strains of Ag–WO_3_ nanoparticles. The Scherrer formula is used to calculate the size of crystallites^125–127^:

4 $${\text{D}} = {\text{k}}\lambda /\left( {\upbeta \;\cos \;\uptheta } \right)$$

where λ is the X-ray wavelength, β is the full width at the half maximum, k is the constant 0.89 < k < 1 change with Miller indices and crystallite shape, but is frequently close to 0.94, and θ is the diffraction angle^128–130^. The dislocation density was calculated using the formula (3) and the Microstrains were determined using Eq. (5). The table demonstrates a small augmentation in crystalline size when the concentration of silver particles is increased. Conversely, the dislocation density and Microstrains exhibited a reduction due to the aforementioned factor. The dislocation density in lines/m2 can be determined by employing the equation^131–133^:

5 $$\updelta = 1/{\text{D}}2_{{{\text{XRD}}}}$$

**Table 3:** XRD parameters of core–shell Ag@WO_3_ nanoparticles prepared with various Ag nanoparticle concentrations.

Ag concentration μg/mL	2θ	FWHM	Miller indices	Crystalline size (nm)	Dislocation density (δ)	Microstrains
0.36	25.5° 58.84°	0.146 0.114	110 220	55.45 63.43	0.078227463 0.038693379	0.062103798 0.043311681
0.76	25.5° 58.84°	0.13 0.093	110 220	56.86 72.82	0.729303684 0.033897991	0.063672181 0.04971665
1.2	25.5° 58.84° 83.4°	0.129 0.089 0.235	110 220 223	62.76 81.25 26.384	0.069118786 0.030207989 0.063461585	0.054872534 0.033813505 0.076545193
1.6	25.5° 58.84° 83.4°	0.121 0.083 0.224	110 220 223	66.91 85.19 27.67	0.064832349 0.02783208 0.06049102	0.052469586 0.031154016 0.072962227
1.96	25.5° 58.84° 83.4°	0.11 0.081 0.211	110 220 223	73.60 89.28 29.38	0.046790533 0.030774089 0.068727812	0.050938499 0.027492664 0.056980402

(d) Lattice strain or Microstrains (η).

The lattice strain is caused by lattice imperfections such dislocations, vacancies, interstitials, and substitutional. These defects cause the atoms to be displaced from their original places in the crystal structure, as a result, the lattice plane d-spacing may be varied. Microstrains will occur during the production of the thin film. This strain may be estimated using the equation below^134–137^.

6 $$\upeta =\upbeta /4\;\tan \left( \Theta \right)$$

The film deposition circumstances will have an impact on structural factors such grain size, crystallinity, and crystal structure.

**Figure 7:**
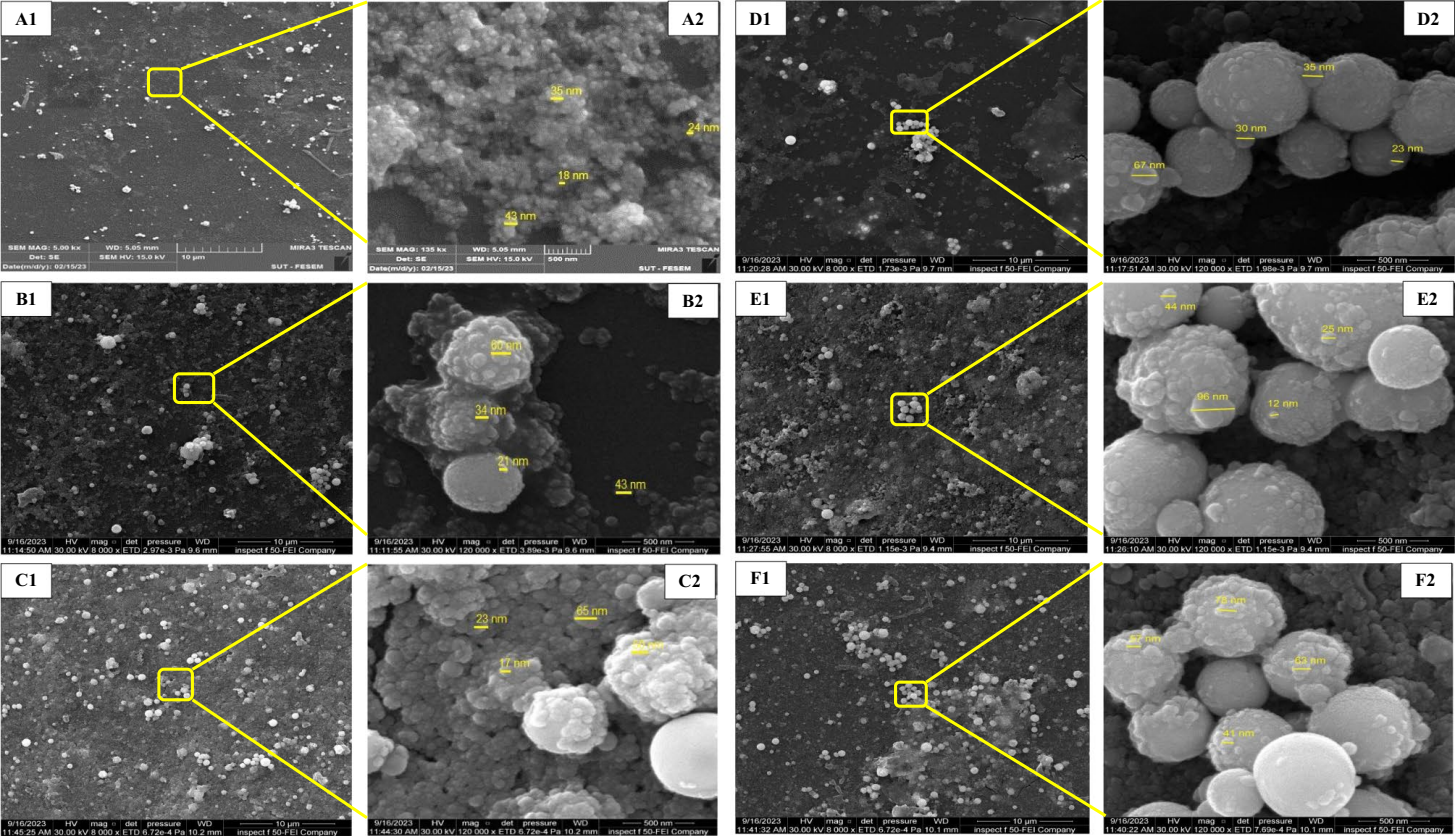
FESEM images of (**a**) WO_3_-NPs and Ag–WO_3_-NPs at various Ag concentrations (**b**—0.36, **c**—0.76, **d**—1.2, **d**—1.6 and **e**—1.96) μg/mL.

Figure 7 shows the FESEM images of Ag–WO_3_ core–shell NPs samples as function of Ag-NPs concentrations. The Ag-NPs concentrations in the samples varied from 0.36 to 1.96 μg/mL. As seen in Fig. 7. The Ag–WO_3_ core–shell structure displays a noticeable augmentation in particle size as the concentration of silver increases. Furthermore, the particles have a well-defined spherical morphology that aligns precisely with the findings of the study^138–140^. The shape, size and agglomerated particles of nanoparticles are determined by the concentrations of Ag-NPs, as seen in Fig. 7. Specifically, the size of WO_3_ outer shell nanoparticles rises proportionally with higher concentrations of Ag-NPs. Additionally, the size of the Ag core also increases in tandem with the Ag-NPs concentrations. This aligns with the findings reported in a prior investigation^141^. The (SEM) images reveal the presence of core/porous-shell structures in the microspheres. The pores inside the shell are densely and uniformly distributed, as seen from the distinct color difference. The shell exhibited a high degree of porosity and was characterized by its minimal thickness. The pictures given below depict various morphological characteristics of both the core and the shell layer. The outer layer, WO_3_, has a higher degree of surface roughness in comparison to the inner layer, Ag.

**Figure 8:**
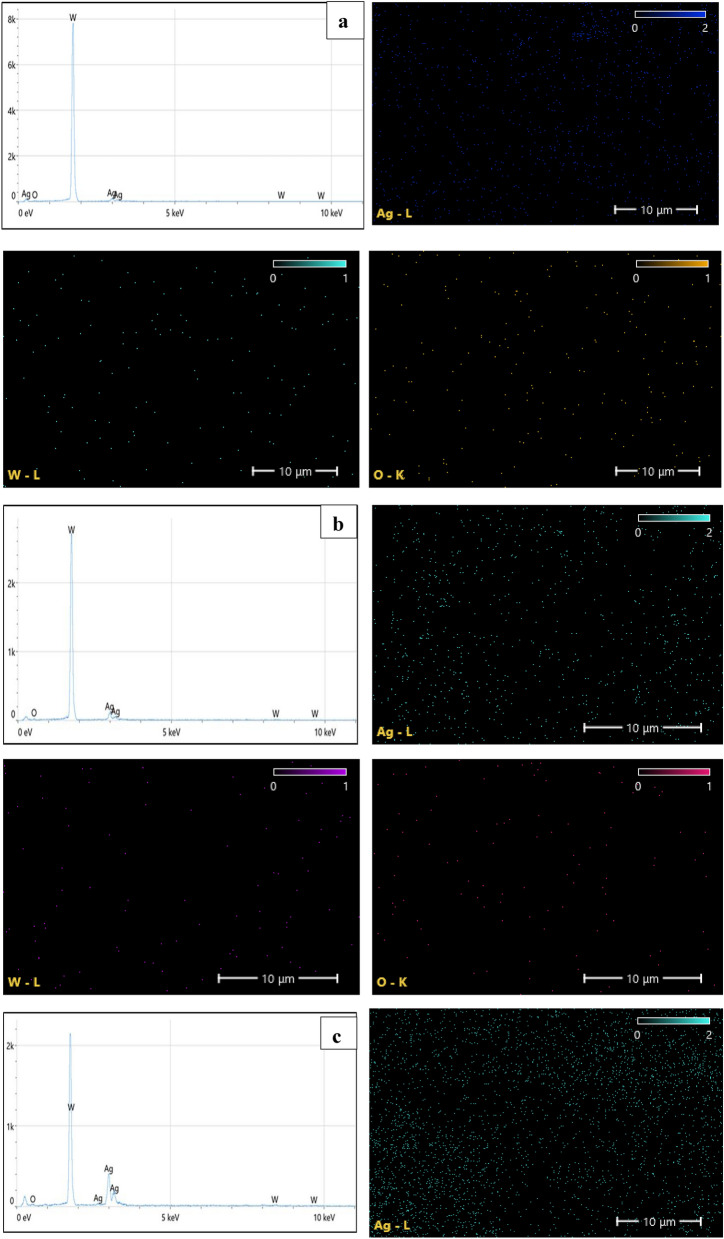

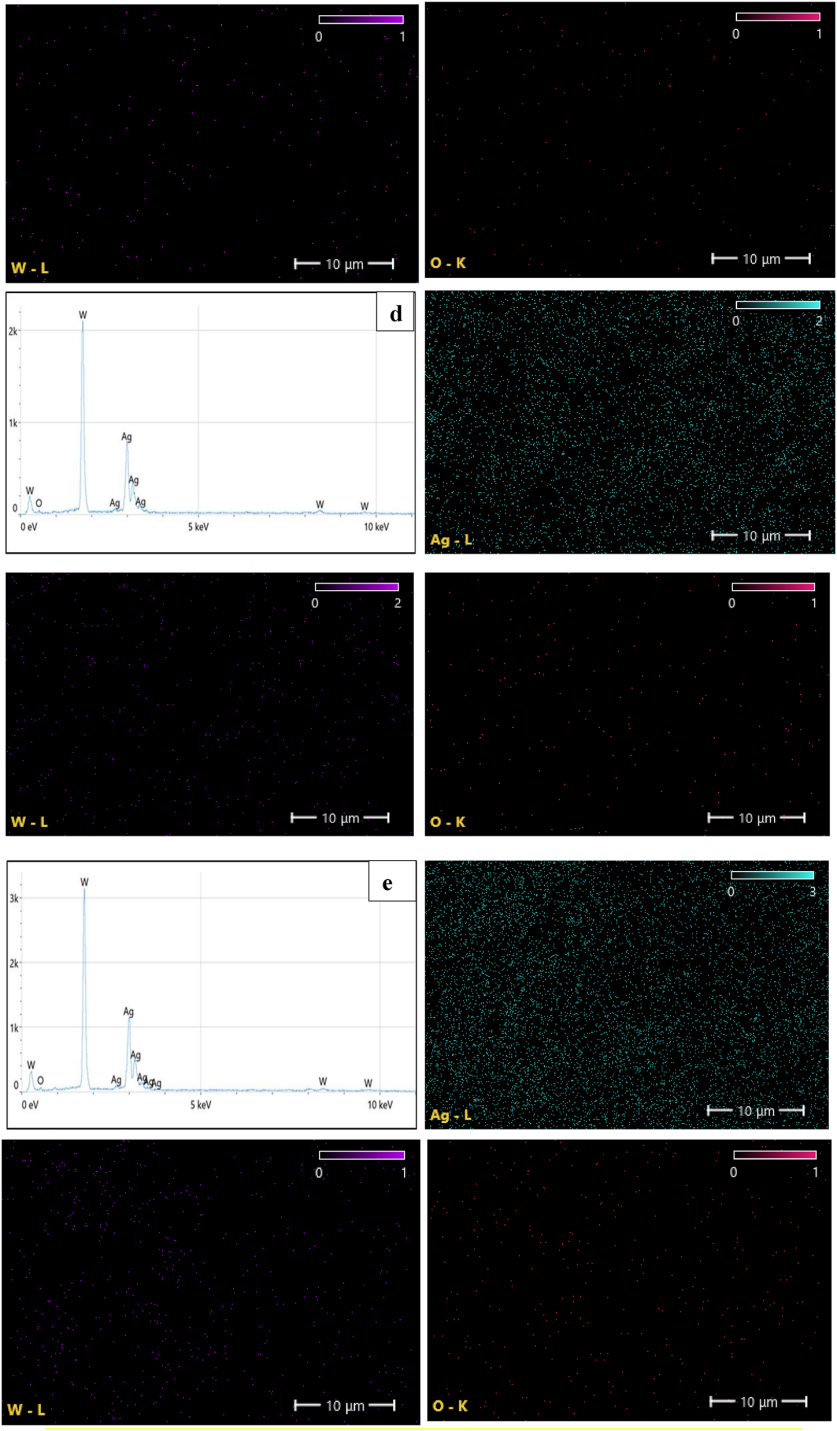
EDX spectra and elemental mapping images for Ag–WO_3_ nanoparticles for various Ag concentrations (**a**—0.36, **b**—0.76, **c**—1.2, **d**—1.6, and **e**—1.96) μg/mL.

The EDX results for Ag–WO_3_ core–shell nanoparticles are depicted in Fig. 8. EDX analysis has the potential to provide insights into the formation process of the Ag–WO_3_ core–shell nanostructure. It can give an indication for the formation of the Ag–WO_3_ core–shell nanostructure, according to Muhammad A. Imam and Nitin Chopra^142,143^. The presence of tungsten (W), oxygen (O), and silver (Ag) in the core shell system was confirmed by this figure. Supporting the EDX results of Ag–WO_3_ core–shell NPs, Fig. 8a,e illustrate the mapping outcomes of Ag, WO_3_, and O, respectively. Ag NPs showed high intensity in EDX due to the increase laser pulses and increasing Ag concentration. Table 4 illustrates the weight percentages of the elements contained in the samples, as well as the stoichiometries of WO_3_. The stoichiometric ratio and weight percentage appear to exhibit an upward trend when the concentration of silver nanoparticles increases.

**Table 4:** Ag–WO_3_-NPs stoichiometry and weight percent of elements.

Ag concentration μg/mL	W %	Ag %	O %	WO_3_ Stoichiometry (%)
0.36	31.2	27.0	41.8	19
0.76	32.2	29.4	38.4	22
1.2	32.6	33.0	34.4	24
1.6	35.0	42.2	22.8	41
1.96	35.2	44.6	20.2	45

**Figure 9:**
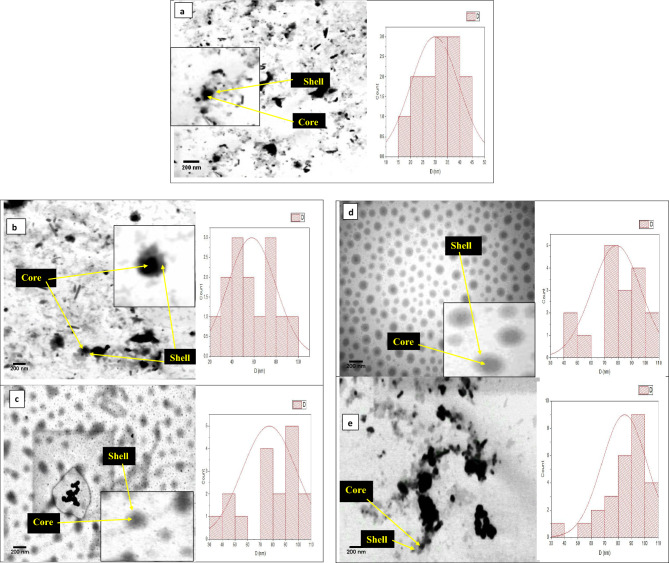
TEM images of Ag–WO_3_-NPs with different Ag concentrations (**a**—0.36, **b**—0.76, **c**—1.2, **d**—1.6, and **e**—1.96) μg/mL.

Figure 9a–e depicts transmission electron microscopy (TEM) pictures of Ag–WO_3_ core–shell NPs samples, illustrating the relationship between the concentration of silver and the observed characteristics. The form and size of NPs are influenced by the concentration of silver, as depicted in the figure. The size of the silver core exhibits a positive correlation with the concentration of silver. In this study, the transmission electron microscopy (TEM) images demonstrate the production of Ag nanoparticles of varying sizes, as well as the presence of some monodisperse Ag particles that are enveloped by a WO3 shell. Additionally, the presence of both aggregated and agglomerated silver particles was noted as increasing the concentration of silver. This matches what was stated in the results of the FE-SEM. Besides, these pictures provide confirmation that the central Ag particles possess spherical morphologies. Table 5 presents data on the average particle size of Ag–WO_3_ core–shell and Ag core. The results indicate that when the concentration of silver nanoparticles was raised from 0.36 to 1.96 μg/mL, the particle size of the Ag–WO_3_ core–shell climbed from 30.5 to 89 nm, while the size of the Ag core increased from 28 to 81 nm. Thus, the manipulation of core size is a viable approach for modulating the overall dimensions of metal-oxide nanoparticles, hence exerting a significant influence on the characteristics of the resulting nanocomposites. This is consistent with what was reported in these studies^144–146^.

**Table 5:** Presents the average particle size of Ag–WO_3_-NPs.

Ag concentration (μg/mL)	Ag core size (nm)	Average particle size of Ag–WO_3_ NPs (nm)
0.36	28	31
0.76	42	45
1.2	60	65
1.6	69	74
1.96	81	89

**Figure 10:**
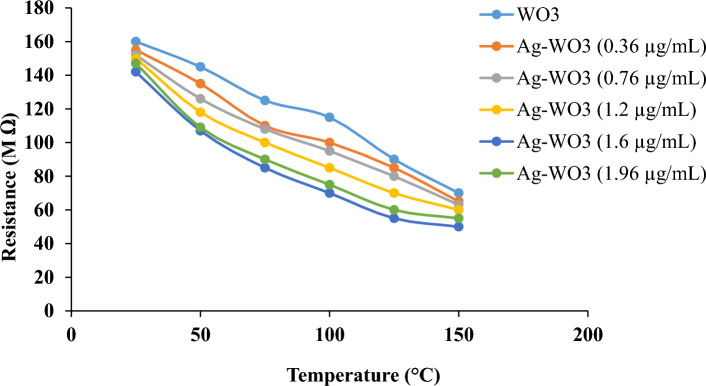
Temperature-dependent resistance of WO_3_ NPs and Ag–WO_3_ core–shell synthesized with various Ag concentrations.

Electrical measurements of WO_3_ NPs and Ag–WO_3_ core–shell samples at various Ag concentrations (0.36, 0.76, 1.2, 1.6, and 1.96 μg/mL) were studied in order to achieve the optimum sample conductivity (lowest resistance). The relation of resistance (R) as a function of temperature (T) was determined, as shown in Fig. 10. The reported resistance values of the Ag–WO_3_ core–shell were high at low temperatures and gradually decreased as the temperature of each sample increased. This is the typical situation for semiconducting materials, as they possess a temperature coefficient that is negative (wherein the resistance decreases as the temperature increases). At thermal equilibrium conditions (room temperature), the initial reading was obtained for each sample to evaluate the resistivity of Ag–WO_3_ manufactured at different Ag concentrations. As shown in this figure, the sample prepared with a concentration of 1.6 g/mL of silver yielded the lowest resistance of approximately 47.1 MΩ at 150 °C. The electrical resistance of the created samples was reduced on a regular basis as compared to the WO3 sample. Because (Ag) nanostructures are metals characterized by elevated levels of free carriers, the conductivity (σ) was raised.

**Figure 11:**
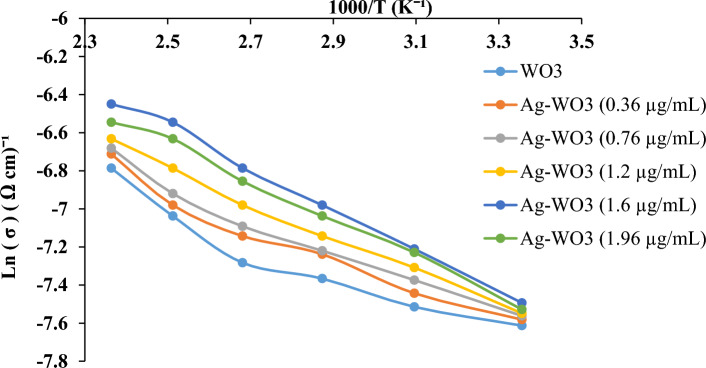
Arrhenius plot curve of ln (σ) versus (1000/T) of WO_3_NPs and Ag–WO_3_ core–shell synthesized with various Ag concentrations.

Figure 11 shows the logarithmic conductivities (lnσ) of the carrier’s curve as a function of reciprocal temperature (1000/T) for WO_3_ and Ag–WO_3_-produced samples with various Ag concentrations (0.36, 0.76, 1.2, 1.6, and 1.96 μg/mL). The corresponding electrical conductivities (σ) of the samples were determined using the equation^147–149^:

7 $$\upsigma = \frac{1}{\uprho }\left( {\updelta \,{\text{cm}}} \right)^{ - 1}$$

The equation shows the resistivity (ρ) in (Ω cm). Based on the figure, the electrical conductivity of the Ag–WO_3_ core–shell increases as the concentration of silver increases. This rise in conductivity was due to an increase in carrier concentration and mobility^150^, whereas the electrical conductivity (σ) values for the sample prepared with 1.96 μg/mL of silver were decreased. This result is due to the aggregation of (Ag) nanostructures in extremely high concentrations, which reduced the carrier mobility. The drop in mobility was directly proportional to the decrease in electrical conductivities^151–153^. This is consistent with what was stated in the previously work^154–156^. Table 6 show the activation energy of Ag–WO_3_ with different Ag concentrations for current work and previous work^156,157^.

**Table 6:** The activation energy of Ag–WO_3_ with different Ag concentrations for current work and previous work.

Current work			Previous work^126^		
Ag concentration (μg/mL)	Average (σdc) (Ω cm)^−1^	Activation energy (eV)	Time (s)	Ag concentration (μg/mL)	Average (σdc) (Ω cm)^−1^
(0 Ag-(WO_3_)	5.89 × 10^−4^	0.155	5	6.328 × 10^−8^	0.222
0.36	7.91 × 10^−4^	0.117	15	8.5624 × 10^−8^	0.221
0.76	8.25 × 10^−4^	0.111	25	1.3619 × 10^−7^	0.188
1.2	8.93 × 10^−4^	0.094	35	3.4491 × 10^−7^	0.220
1.6	10.6 × 10^−4^	0.091	45	1.8557 × 10^−7^	0.192
1.96	9.91 × 10^−4^	0.084			

The Arrhenius relation was used to calculate the activation energy^158–160^:

8 $$\upsigma =\upsigma ^\circ \;{\text{exp}}\left( { - {\text{Ea/KT}}} \right)$$

where the conductivities of carriers are denoted by the symbol σ, σ° represents the temperature of the independent portion conductivity, Ea denotes the necessary amount of energy for activation, then tn, constant of Boltzman (K), and the temperature, denoted by the letter T, is expressed as a value in Kelvin (k) unit. The activation energy values as a function of Ag concentration in the Ag–WO_3_ samples are listed in Table 6.

**Figure 12:**
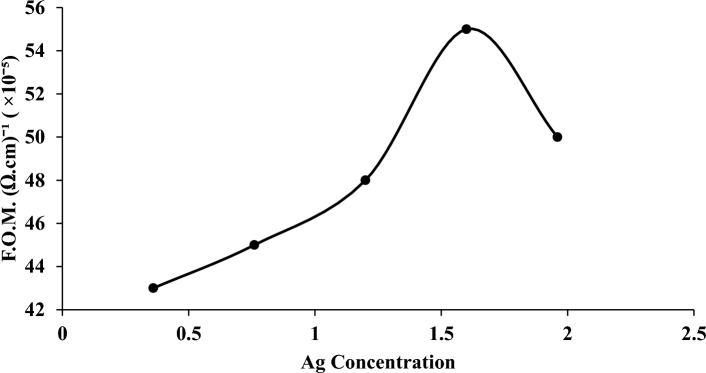
F.O.M. of Ag–WO_3_ core–shell synthesized with various Ag concentrations.

The figure of merit (F.O.M.) of Ag–WO_3_ core–shell samples created with various silver concentrations is displayed in Fig. 12. They were computed using Eq. (9) which was provided by Iles and Soclof^161–163^:

9 $${\text{FOM}} = \frac{\upsigma }{\upalpha }$$

The absorption coefficient is represented by the symbol (α) and is the conductivity of electricity. The requirement for calculating the F.O.M. was to realize the optimal electrical conductivity (σ) as a function of the absorption coefficient (α) of Ag–WO_3_ that was made by varying the amount of Ag (0.36, 0.76, 1.2, 1.6, and 1.96 μg/mL). The concentration of 1.6 μg/mL of silver used in the preparation of the Ag–WO_3_ core shell is considered a merit figure, as the incorporation of silver (Ag) was intended to improve the semiconductor material's properties.

## Conclusion

We are successively synthesizing a Novel Ag–WO_3_ core–shell nanostructure using a two-step laser ablation process in deionized water. The impact of varying core concentrations (Ag-NPs) on the structural, electrical, morphological, and optical characteristics of Ag–WO_3_ core–shell NPs have been investigated. XRD analysis has confirmed the growth of a hexagonal polycrystalline WO_3_ phase and the creation of cubic Ag-NPs. The presence of Ag–WO_3_ core–shell NPs was verified using the XRD profile, which exhibited prominent diffraction planes. The transmission electron microscopy (TEM) analysis demonstrated that the size of the Ag core and the thickness of the WO_3_ shell were dependent on the concentration of the core (Ag). Additionally, the study showed that the Ag–WO_3_ nanoparticles exhibited a spherical morphology. The photoluminescence (PL) data exhibited a wide peak with a central wavelength of 456 nm. Following the creation of a hybrid Ag–WO_3_ core–shell structure, the PL intensity of WO_3_ was quenched. The wavelength of the absorption peak was abundantly evident at (414–417) nm. As a result of the increased ablation, the absorbance rose as the Ag-NPs concentration increased. A substantial decrease in the band gap was observed, with a transition from 2.73 to 2.5 electron volts (eV). The DC conductivity exhibited a distinctive semiconductor-like behavior, showing an increase with elevated temperatures. The figure of merit (F.O.M.) with the best performance was identified by an electrical conductivity observed at a concentration of 1.6 μg/mL of silver. The electrical conductivity of Ag–WO_3_ has seen a notable increase, with the most advantageous characteristics due to the incorporation of Ag-NPs.

## Data Availability

Correspondence and requests for materials should be addressed to Evan T. Salim, Maryam S. Muhsin, Makram A. Fakhri.
